# Predictors of breast milk substitute feeding among newborns in delivery facilities in urban Cambodia and Nepal

**DOI:** 10.1111/mcn.12754

**Published:** 2019-06-21

**Authors:** Mary Champeny, Alissa M. Pries, Kroeun Hou, Indu Adhikary, Elizabeth Zehner, Sandra L. Huffman

**Affiliations:** ^1^ Helen Keller International New York NY USA; ^2^ London School of Hygiene and Tropical Medicine London UK

**Keywords:** breast milk substitutes, breastfeeding, breastfeeding initiation, infant feeding behaviour, infant feeding decisions, infant formula

## Abstract

Introducing breast milk substitutes (BMS) in the first days after birth can increase infant morbidity and reduce duration and exclusivity of breastfeeding. This study assessed determinants of BMS feeding among newborns in delivery facilities in Phnom Penh, Cambodia, and Kathmandu Valley, Nepal. Cross‐sectional surveys were conducted among mothers upon discharge from health facilities after delivery: 304 mothers in Kathmandu Valley and 306 mothers in Phnom Penh participated. On the basis of a conceptual framework for prelacteal feeding, multivariable logistic regression was used to identify factors associated with BMS feeding prior to facility discharge. In both Phnom Penh and Kathmandu Valley, feeds of BMS were reported by over half of mothers (56.9% and 55.9%, respectively). Receiving a health professional's recommendation to use BMS increased the odds of BMS feeding in both Kathmandu Valley and Phnom Penh (odds ratio: 24.87; confidence interval [6.05, 102.29]; odds ratio: 2.42; CI [1.20, 4.91], respectively). In Kathmandu Valley, recommendations from friends/family and caesarean delivery were also associated with BMS use among mothers. Early initiation of breastfeeding and higher parity were protective against the use of BMS in Kathmandu Valley. Breastfeeding support from a health professional lowered the odds of BMS feeding among newborns. Exposure to BMS promotions outside the health system was prevalent in Phnom Penh (84.6%) and Kathmandu Valley (27.0%) but was not associated with BMS feeds among newborns. Establishment of successful breastfeeding should be prioritized before discharging mothers from delivery facilities, and health professionals should be equipped to support and encourage breastfeeding among all new mothers.

**Key messages**

- Breast milk substitute (BMS) feeding before discharge from delivery facilities among healthy, term, singleton births is common in Phnom Penh and Kathmandu Valley.
- Health worker recommendations for mothers to use BMS are strongly associated with early BMS feeding.
- Mothers who initiate breastfeeding within an hour of birth in Kathmandu Valley are less likely to introduce BMS before leaving the delivery facility. Those who receive breastfeeding positioning or attachment support from health professionals in Phnom Penh are also less likely to introduce BMS to their infant while in the delivery facility.
- In Kathmandu Valley, caesarian delivery is highly associated with BMS use; to improve their likelihood of initiating and sustaining breastfeeding of newborns, better support for these mothers is needed, including encouraging immediate skin‐to‐skin contact.
- Despite legislation restricting promotion of BMS, mothers in both countries recalled observing commercial promotions both inside and outside the health system.

## 1 INTRODUCTION

Breastfeeding has a protective effect against infant morbidity and mortality (Victora et al., 2016) and reduces the risk of several non‐communicable diseases later in life (Kørvel‐Hanquist, Djurhuus, & Homøe, 2017; Morris, 2018; Wang, Collins, Ratliff, Xie, & Wang, 2017). There are benefits to the mother, in terms of reduced risks of breast cancer, ovarian cancer, endometriosis, and type 2 diabetes, as well as increased birth spacing (Farland et al., 2017; Victora et al., 2016). Colostrum, the mother's first milk produced from birth until about day 3 of life, is a unique bioactive substance, which has important immunologic, nutritional, and developmental functions in the newborn (Andreas, Kampmann, & Mehring Le‐Doare, 2015). Providing an infant with anything other than breast milk in the first few days after birth (prelacteal feeding) is associated with delayed initiation of breastfeeding (Grassley, Schleis, Bennett, Chapman, & Lind, 2014; Sharma & Byrne, 2016) and increases risk of pathogenic infection and diarrheal disease (Debes et al., 2013; Ogbo, Page, Idoko, Claudio, & Agho, 2016). In‐hospital breast milk substitute (BMS) supplementation during this period has a negative impact on later exclusive and continued breastfeeding (Chantry, Dewey, Peerson, Wagner, & Nommsen‐Rivers, 2014; T. T. Nguyen, Withers, Hajeebhoy, & Frongillo, 2016; Vehling et al., 2018).

Despite the recognized health impacts, only 28% of infants born in low‐ and middle‐income countries (LMIC) begin breastfeeding within an hour of birth and receive only breast milk in the first 3 days of life (Oakley, Benova, Macleod, Lynch, & Campbell, 2017). These same countries bear a disproportionate burden of the world's maternal and child mortality, as well as low birthweight, stunting, and wasting (United Nations Children's Fund [UNICEF], 2016). Universal optimal breastfeeding could save 823,000 child lives and 20,000 maternal lives each year (Victora et al., 2016). In Southeast Asia, health expenditure costs attributed to not breastfeeding amount to nearly $300 million USD each year (Walters et al., 2016).

Women living in urban areas within Asia, including Cambodia and Nepal, are now more likely to deliver their babies in health facilities than at home, representing a major shift over the past decade (Pomeroy, Koblinsky, & Alva, 2014), which is consistent with trends observed across LMIC settings (Campbell et al., 2016; Munabi‐Babigumira, Glenton, Lewin, Fretheim, & Nabudere, 2017). In these contexts, the health system serves as a critical source of breastfeeding advice and support in the early moments after delivery.

The market for BMS is large and growing, with global sales in 2014 reaching over $44 billion USD (Rollins et al., 2016). South and Southeast Asia in particular have seen explosive growth in BMS sales, where breastfeeding rates are also in rapid decline (Baker et al., 2016). Promotion and consumption of these products have increased in step with global sales volume (Kent, 2015). Marketing of BMS within the health care system undermines mothers' confidence in breastfeeding (Parry, Ip, Chau, Wu, & Tarrant, 2013) and has been observed across South and Southeast Asia even when national legislation is in place to limit it (Barennes, Slesak, Goyet, Aaron, & Srour, 2016; Durako, Diallo, Thompson, & Aronson, 2016a; Durako, Diallo, Thompson, & Aronson, 2016b; Hidayana et al., 2016; Sobel et al., 2011). Prior research has shown that early use of BMS for feeding of newborns can increase the likelihood of continued BMS use for infant feeding and contribute to early breastfeeding cessation (Onah et al., 2014; Patil et al., 2015; Raheem, Binns, Chih, & Sauer, 2014).

There is a need for more information on the factors that influence newborn feeding in health facilities following the birth of a child, because experiences in the immediate post‐partum period have implications for how children are fed throughout infancy. Few studies of early in‐hospital BMS use in urban LMIC Asian contexts seek to assess the influence of BMS promotion on feeding before discharge after the delivery of a newborn, together with factors related to the birth experience and post‐partum health facility environment. The aim of this study was to explore socio‐demographic characteristics, delivery characteristics, and marketing exposure factors associated with BMS feeding of newborns among mothers in Phnom Penh, Cambodia, and Kathmandu Valley, Nepal. Specific objectives included assessment of mothers' exposure to promotional practices for commercial infant and young child feeding products and BMS consumption among newborns within delivery facilities.

## 2 METHODS

### 2.1 Study design and sampling

Structured interviews were conducted as part of cross‐sectional, health facility‐based surveys among mothers being discharged after delivery of their newborns in Phnom Penh, Cambodia, and Kathmandu Valley, Nepal. Interviews were conducted in‐person at health facilities from November 2013–February 2014. A sample size of 280 mothers discharged after delivery was required in each city in order to detect a 10% prevalence of mothers exposed to BMS promotions within the health system, with a measurement error of ±5, standard of error of 0.0255, and assuming a design effect of 2 to account for multistage cluster sampling (Pries, Huffman, & Champeny, 2015; Pries, Huffman, Mengkheang, et al., 2016). A multistage cluster sampling strategy was used to obtain a representative sample of mothers delivering in each city's health system. Each cluster included 16 mothers in order to have sufficient variation in facilities, and 19 clusters were selected to have 304 in total, slightly more than needed. Because sampling of facilities was proportional to size, larger facilities had a greater chance of being sampled for multiple clusters. Approximately 30% of urban Cambodian mothers with facility‐based deliveries deliver at private facilities (National Institute of Statistics, Directorate General for Health, & ICF Macro, 2011); therefore, 30% of discharged mothers (six clusters with 16 mothers each) were interviewed at private facilities.

In Phnom Penh, Cambodia, lists of all public health facilities offering public maternity services and utilization numbers were obtained from the Health Management Information System database of the Ministry of Health for 2012, the year preceding the survey. This included national hospitals, referral hospitals, and health centres; health posts were excluded. Due to the need to complete data collection within 8–10 weeks, facilities with less than 50 deliveries per month were excluded from the sampling frame. This excluded 27 out of 36 public facilities for delivery, but the nine facilities included in the final sampling frame represented 79.6% of all public facility‐based births in Phnom Penh in January–December 2012.

Because a complete listing and utilization rates of private maternity facilities was not available, a comprehensive list was developed on the basis of input from local experts and scoping of the private health facility landscape in Phnom Penh. Each private facility identified was contacted in order to request monthly delivery numbers, and these were cross‐checked by a repeated request for information several weeks later. Twenty‐six private maternity facilities in Phnom Penh were contacted. Of these, only one reported rates of 50 or more deliveries per month on average. Therefore, the remaining 25 facilities were randomly assigned to form five groupings consisting of 3 to 8 facilities each so that their cumulative monthly delivery rates were at least 50. Thus, nine public facilities were included in the public sampling frame, and seven facilities were included in 13 selected clusters using probability proportional to size sampling. Out of the 26 private facilities, 22 (from four groupings and the one facility with more than 50 deliveries per month) were included in six randomly selected clusters.

In Kathmandu Valley, information on both public and private hospital deliveries was available through government statistics, and these were included as part of the sample, with 30% of the sample also from private hospitals. Facilities with less than 50 deliveries per month were excluded from the sampling frame, resulting in 40 out of 48 facilities that represented an estimated 91.0% of facility‐based deliveries in Kathmandu Valley. The sample was proportional to the number of births delivered in public and private hospitals. Pries, Huffman, Adhikary, et al. (2016) gives details on the sample selection for Kathmandu Valley.

### 2.2 Study population

In Phnom Penh, 441 mothers being discharged after delivery were approached for interview, and 108 were excluded because they lived outside of Phnom Penh. Of the remaining 333 mothers, 7 refused participation and 20 were excluded on the basis of at least one of the criteria detailed here: 17 mothers reported severe complications during delivery, 11 infants had been in the neonatal intensive care unit after delivery, and 2 children were from a multiple birth. The final sample of successfully completed interviews among mothers discharged after delivery was 306 mothers.

In Kathmandu Valley, 452 mothers were approached, and 101 were excluded because they resided outside the city. Of the remaining 351, 26 refused participation and 21 mothers were excluded on the basis of at least one of the following criteria: 20 infants had been in the neonatal intensive care unit after delivery, 8 mothers reported severe complications during delivery, and 2 children were from a multiple birth. In Kathmandu Valley, a total of 304 mothers were interviewed.

Data were gathered regarding mothers' socio‐demographic characteristics, type of delivery, and feeding practices (including initiation of breastfeeding, prelacteal feeding, and current breastfeeding) before discharge from delivery facilities. Mothers were also asked to report on support received during antenatal care as well as during delivery and initiation of breastfeeding, any recommendations to use BMS or other foods/liquids, and whether they had observed commercial promotions for BMS inside and outside of the health system. Commercial promotions were defined as any type of marketing activity meant to increase sales of a BMS product including but not limited to: media or print advertising, provision of free samples or branded gifts, branded equipment in use at health facilities, and retail promotions (World Health Organization & UNICEF, 2017). Ethics approval for this study was obtained through the Cambodia National Ethics Committee for Health Research and the Nepal Health Research Council. Written informed consent was collected from all participants prior to interview.

### 2.3 Variable definitions

**Figure 1:**
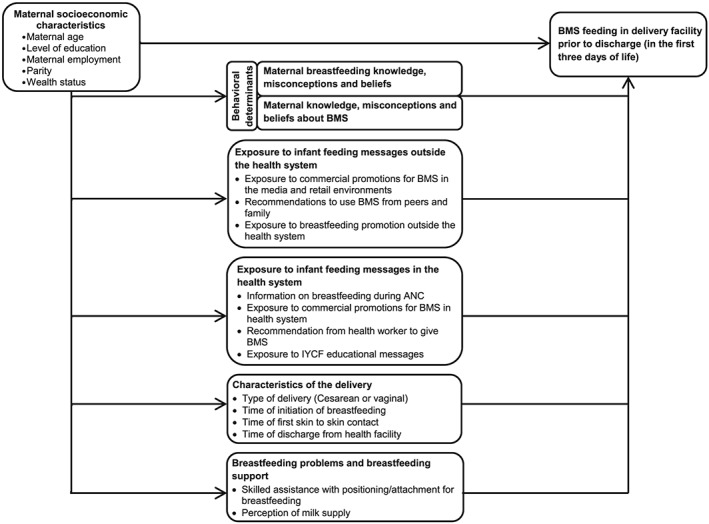
Conceptual framework for determinants of feeding of breast milk substitutes to newborns in health facilities prior to discharge, adapted from P. H. Nguyen et al. (2013). ANC: antenatal care; BMS: breast milk substitute

A conceptual framework adapted from P. H. Nguyen et al. (2013) was used to identify theoretical covariates of BMS feeding prior to discharge from delivery facilities. Covariate variables were grouped into five categories: (a) maternal socio‐economic characteristics, (b) exposure to infant feeding messages outside the health system, (c) exposure to infant feeding messages inside the health system, (d) characteristics of the delivery, and (e) breastfeeding problems and support; details of covariate variables included for each category are shown in Figure 1.

To determine educational attainment, mothers were asked to report the highest level of schooling they attended. A wealth index based on principal components analysis (Vyas & Kumaranayake, 2006) was used to create wealth terciles for each sample. Variables in this index included access to safe drinking water, household crowding, access to electricity, home ownership, and ownership of a mobile phone, television, refrigerator, bicycle, motorbike, or car. Binary variables were created for categorical variables, with women's educational attainment at the university level compared with all women in lower educational categories and those in the highest wealth tercile compared with women in the lower two terciles. Mothers' employment was categorized as paid work taking place inside or outside the home.

The recall period for questions of exposure to infant feeding messages both inside and outside the health system was during the mother's most recent pregnancy or since the birth of her child. To assess exposure to commercial promotions, mothers were asked if they had heard, seen, or read any commercial promotions, and those who responded “yes” were asked to recall the locations where they had seen promotions. These locations were later coded as inside or outside the health system. A similar protocol was used for exposure to breastfeeding promotion. Mothers were first asked to report whether they had read, seen, or heard “any educational messages or information on infant and young child feeding” and then asked to recall any locations associated with these messages. Mothers were then asked to describe the content of these messages, and those which dealt with the importance of colostrum feeding, early initiation of breastfeeding, exclusive breastfeeding until 6 months or continued breastfeeding to 2 years were coded as breastfeeding promotions. Mothers were asked if they received any recommendations to use BMS during pregnancy or after birth and then asked to report the source of these recommendations; those coming from health workers (including doctors, nurses, midwives, dietitians, and other health professionals) were considered exposure to infant feeding messages in the health system, whereas reported recommendations from peers and family (including friends, neighbours, or relatives) were considered exposure to infant feeding messages outside the health system.

Initiation of breastfeeding was coded as early (within 1 hr after birth) or delayed (more than 1 hr after birth). Information on skin‐to‐skin contact after birth was not collected directly; instead, mothers were asked to report the duration of time from birth until she first held her infant, which was used as a proxy indicator. “Skilled assistance” was defined as assistance with positioning or attachment for breastfeeding received from a health worker at any time before discharge from the health facility. Length of stay in the health facility after delivery was categorized as discharge less than 3 days after birth or discharge more than 3 days after birth. The survey did not assess behavioural determinants of BMS feeding, including maternal knowledge, misconceptions, and beliefs (Figure 1).

The outcome of interest was mothers' use of BMS for newborn feeding in the first 3 days of life while in the health facility. To assess this, mothers were first asked if their newborn was given anything to drink other than breast milk in the first 3 days after delivery. Those who answered “yes” were then asked to list what specific liquids or foods were given and asked to describe the reason the child received the feed. All who listed BMS were coded as providing a BMS feed after delivery.

Prelacteal feeding is generally defined as the introduction of foods or liquids other than breast milk in the first 3 days after delivery (Mukuria, Kothari, & Abderahim, 2006). This study's recall period makes use of the term “prelacteal feeding” unsuitable to describe the introduction of BMS among this sample. The length of stay after delivery ranged from less than 24 hr to over 9 days, meaning that complete data from the first 3 days after birth was not available for all mothers. Additionally, prelacteal feeding, along with other terms like “in‐hospital formula supplementation” used in similar studies, is often calculated only among mothers who ever breastfed (Chantry et al., 2014; P. H. Nguyen et al., 2013; Parry, Ip, et al., 2013; Tarrant et al., 2015). In the present study, the use of BMS and other foods was evaluated among all mothers, whether or not they ever breastfed, and adjusted models controlled for mothers' length of stay in the health facility.

### 2.4 Statistical analysis

Data were cleaned using SPSS version 21 (IBM Corp, 2017) and analysed using Stata 15 (StataCorp, 2017). Initial descriptive statistics were conducted, and proportions, means, and standard deviations were calculated to present characteristics of the sample. Bivariate analyses were conducted to detect differences in proportions using two‐sided Pearson chi‐square tests and differences in means using independent *t* tests. Multivariable logistic regression models were developed for each sample to assess the theoretical covariates of mothers' reported use of BMS to feed their newborn while in the hospital, with odds ratios and 95% confidence intervals (CIs) generated for each covariate variable in both unadjusted and adjusted analyses. Models used generalized estimating equations with robust standard errors to account for clustering at the healthy facility levels. Child sex was included because of the potential for sex bias in use of commercial infant and young child feeding products, but its inclusion/exclusion had no impact on model fit. Model fit was determined through the quasi likelihood under independence model criterion, and collinearity of covariates included in the models was explored through variance inflation factors.

## 3 RESULTS

### 3.1 Characteristics of the sample

**Figure 2:**
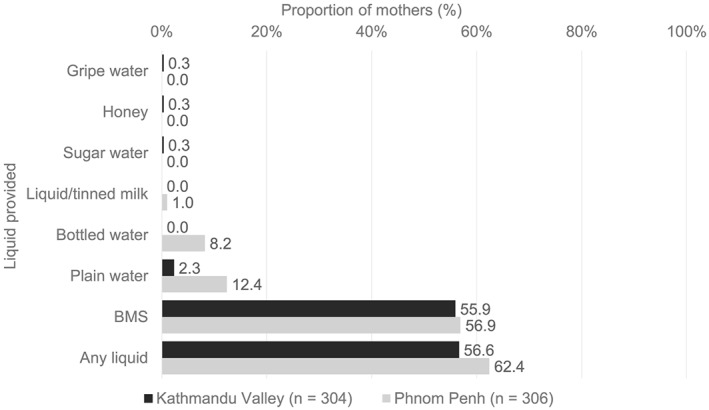
Proportion (%) of mothers who reported providing liquids to their newborn before discharge after delivery, by type. BMS: breast milk substitute

**Figure 3:**
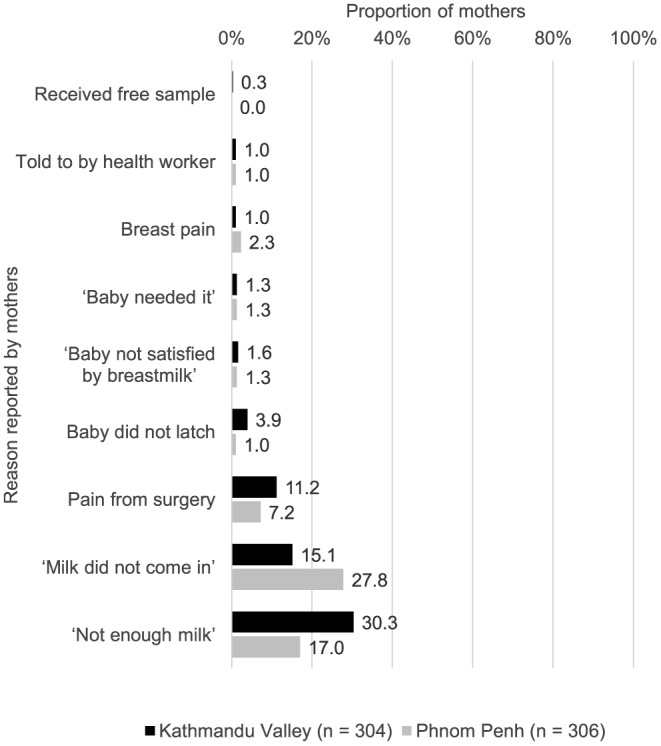
Mothers' reported reasons for providing breast milk substitutes to their newborn before discharge from health facilities

The study populations in each site were similar (Table 1). Most respondents were married (99.7% in Kathmandu Valley and 99.3% in Phnom Penh), had more than one child on average, and the majority of mothers attended secondary school or had higher education. Socio‐demographic characteristics of the mothers included in this study along with relative frequencies of those mothers who reported feeding BMS to their newborn before discharge and those who did not are shown in Table 1. Over half of mothers in both sites provided foods or liquids besides breast milk before being discharged after delivery, most commonly BMS (Figure 2). When asked why a BMS was provided, mothers' most commonly reported reasons were concerns that their milk supply was insufficient or had not yet “come in” (Figure 3).

**Table 1:** Demographics and socio‐economic characteristics by use of BMS among newborns

	Kathmandu Valley			Phnom Penh		
	Total (*n* = 304)	Provided BMS (*n* = 170)	No BMS provided (*n* = 134)	Total (*n* = 306)	Provided BMS (*n* = 174)	No BMS provided (*n* = 132)
Maternal						
Age (years)	25.0 ± 4.6	25.4 ± 4.7	24.4 ± 4.4	27.3 ± 4.7	27.6 ± 4.6	26.9 ± 4.9
Parity (number)	1.5 ± 0.6	1.3 ± 0.6	1.6 ± 0.7***	1.7 ± 1.0	1.6 ± 0.8	1.8 ± 1.2*
Level of education						
No education/primary only	29.6 (90)	23.5 (40)	37.7 (50)**	35.0 (107)	28.2 (49)	43.9 (58)**
Secondary	51.3 (156)	49.4 (84)	53.7 (72)	43.8 (134)	46.6 (81)	40.2 (53)
Tertiary	19.1 (58)	27.1 (46)	9.0 (12)***	21.2 (65)	25.3 (44)	15.9 (21)*
Works outside the home^a^	15.1 (46)	18.8 (32)	10.5 (14)*	30.4 (93)	32.8 (57)	27.3 (36)
Sex of infant (male)^a^	56.3 (171)	58.8 (100)	53.0 (71)	48.7 (149)	48.3 (84)	49.2 (65)
Household						
Wealth terciles						
Lowest tercile	35.9 (109)	29.4 (50)	44.0 (59)**	23.9 (73)	14.9 (26)	35.6 (47)***
Middle tercile	30.9 (94)	28.8 (49)	33.6 (45)	44.8 (137)	46.0 (80)	43.2 (57)
Highest tercile	33.2 (101)	41.8 (71)	22.4 (30)***	31.4 (96)	39.1 (68)	21.2 (28)**

*Note*. Data presented as % (*n*) or mean ± standard deviation; proportions compared using Pearson chi‐square test, means compared using independent *t* tests. Comparisons between providers and non‐providers of BMS, by site.

BMS: breast milk substitute.

a Binary categorical variable.

* *P* ≤ 0.05.

** *P* ≤ 0.01.

*** *P* ≤ 0.001.

Mothers' reported delivery characteristics, exposure to infant feeding messages, and other theorized covariates of BMS use before facility discharge based on the conceptual framework are presented in Table 2.

**Table 2:** Theoretical covariates for use of BMS among newborns prior to health facility discharge, by study site

	Kathmandu Valley			Phnom Penh		
	Total (*n* = 304)	Provided BMS (*n* = 170)	No BMS provided (*n* = 134)	Total (*n* = 306)	Provided BMS (*n* = 174)	No BMS provided (*n* = 132)
Exposure to infant feeding messages outside the health system						
Observed BMS promotion in media or retailer	27.0 (82)	28.2 (48)	25.4 (34)	84.6 (259)	87.9 (153)	80.3 (106)
Recommendation from peers/family to use BMS	13.5 (41)	18.8 (32)	6.7 (9)**	22.5 (69)	25.3 (44)	18.9 (25)
Exposure to IYCF educational messages in mass media	19.1 (58)	24.1 (41)	12.7 (17)*	41.5 (127)	41.4 (72)	41.7 (55)
Exposure to infant feeding messages inside the health system						
Observed BMS promotion inside a health facility	7.9 (24)	8.2 (14)	7.5 (10)	45.1 (138)	45.4 (79)	44.7 (59)
Recommendation from health professional to use BMS	47.4 (144)	74.7 (127)	12.7 (17)***	13.4 (41)	17.2 (30)	8.3 (11)*
Exposure to IYCF educational messages inside health system	18.4 (56)	18.2 (31)	18.7 (25)	43.8 (134)	39.1 (68)	50.0 (66)
Received information on breastfeeding during ANC	11.5 (35)	5.9 (10)	18.7 (25)**	36.3 (111)	34.5 (60)	38.6 (51)
Characteristics of the delivery						
Caesarean delivery	29.3 (89)	47.1 (80)	6.7 (9)***	23.5 (72)	31.6 (55)	12.9 (17)***
Early initiation of breastfeeding	40.8 (124)	28.8 (49)	56.0 (75)***	34.0 (104)	21.8 (38)	50.0 (66)***
Held newborn immediately after delivery	8.6 (26)	7.7 (13)	9.7 (13)	55.6 (170)	46.6 (81)	67.4 (89)***
Breastfeeding support						
Received positioning/attachment assistance for breastfeeding	63.8 (194)	69.4 (118)	56.7 (76)*	57.5 (176)	50.6 (88)	66.7 (88)**

*Note*. Data presented as % (*n*); proportions compared using Pearson chi‐square tests. Theoretical covariates based on feeding of breast milk substitutes to newborns in health facilities prior to discharge. All variables are binary categorical.

ANC: antenatal care; BMS: breast milk substitute; IYCF: infant and young child feeding.

* *P* ≤ 0.05.

** *P* ≤ 0.01.

*** *P* ≤ 0.001.

### 3.2 Exposure to infant feeding messages outside the health system

There were substantial differences in mothers' reported exposure to commercial promotions for BMS during pregnancy (Table 2). In Phnom Penh, 84.6% (*n* = 259) of mothers recalled observing commercial promotions for BMS outside health facilities with 80.7% (*n* = 247) reporting observing these promotions through television and 21.9% (*n* = 67) seeing promotions in shops or pharmacies. Over one fourth (27.0%, *n* = 82) of mothers in Kathmandu Valley reported exposure to such marketing, again, mostly through television (21.4%, *n* = 65).

Recommendations to use BMS from the mother's peer groups or family members during her pregnancy or after delivery were reported by slightly more Phnom Penh mothers (22.5%, *n* = 69) than Kathmandu Valley mothers (13.5%, *n* = 41). Educational messages about infant and young child feeding distributed through mass media channels, including television, radio, internet, mobile messaging, and printed materials, were also more frequently reported by Phnom Penh mothers. These mothers most commonly reported messages about introduction of complementary foods at 6 months of age. In contrast, Kathmandu Valley mothers who were exposed to such mass media messages most commonly reported hearing information related to exclusive breastfeeding.

### 3.3 Exposure to infant feeding messages inside the health system

In Kathmandu Valley, recommendations to use BMS during pregnancy from any source were reported by 56.9% (*n* = 173) of mothers. These recommendations frequently came from health professionals (Table 2). Nurses, midwives, and other health professionals were more commonly cited as the source of these recommendations (38.8%, *n* = 118) than doctors (8.6%, *n* = 26). A greater proportion of mothers in both sites who received health professional recommendations provided BMS in the delivery facility (Kathmandu Valley *P* ≤ 0.001, Phnom Penh *P* ≤ 0.05) compared with those who reported no recommendation. Compared with their Kathmandu Valley counterparts, more Phnom Penh mothers reported observing commercial promotions for BMS within a health care facility (Table 2), mostly in the form of branding or logos on health facility equipment (31.7%, *n* = 97). No mothers in Kathmandu Valley reported receiving samples of BMS or bottle‐feeding supplies in health facilities as part of these commercial promotions, whereas 9.8% (*n* = 30) of Phnom Penh mothers did.

Educational messages about infant and young child feeding distributed through health facilities were more commonly recalled by Phnom Penh mothers compared with those in Kathmandu Valley (Table 2). In Phnom Penh, these messages were primarily about introduction of complementary foods at 6 months of age (26.8%, *n* = 82) and exclusive breastfeeding (23.9%, *n* = 73), whereas in Kathmandu Valley, 11.8% (*n* = 36) of mothers reported exposure to messages about the importance of exclusive breastfeeding in health facilities.

### 3.4 Characteristics of the delivery and breastfeeding practices

Less than half of mothers initiated breastfeeding within 1 hr after birth. Upon discharge from the delivery facility, 89.2% (*n* = 273) of Phnom Penh mothers and 96.1% (*n* = 292) of Kathmandu Valley mothers reported currently breastfeeding their babies (defined as any breastfeeding in the past 24 hr). Over 60% (63.7%, *n* = 195) of mothers in Phnom Penh stayed 3 days or more in the delivery facility before discharge. In Kathmandu Valley, 56.9% (*n* = 173) of mothers were discharged between 24–28 hr after delivery, and an additional third (31.6%, *n* = 96) of mothers were discharged after 3 days. Rates of caesarean delivery were comparable between Kathmandu Valley and Phnom Penh mothers, at 29.3% (*n* = 89) and 23.5% (*n* = 72), respectively. The vast majority of mothers who delivered by caesarian had a hospital stay of more than 3 days after delivery: 92.1% (*n* = 82) in Kathmandu Valley and 98.6% (*n* = 71) in Phnom Penh. In both sites, as compared with mothers who had vaginal deliveries, a greater proportion of mothers delivering by caesarean provided BMS. A smaller proportion of mothers who initiated breastfeeding within 1 hr provided BMS as compared with mothers who initiated breastfeeding after 1 hr (Table 2).

### 3.5 Breastfeeding support

Nearly all Kathmandu mothers (99.3%, *n* = 302) and Phnom Penh mothers (87.9%, *n* = 269) reported receiving antenatal care from a health provider during pregnancy. Few mothers reported having received breastfeeding education or information during antenatal care visits in Kathmandu Valley, whereas this was reported among over a third of Phnom Penh mothers. In Phnom Penh, the antenatal care messages most commonly recalled were on the importance of exclusive breastfeeding (19.3%, *n* = 59) and early initiation of breastfeeding (19.0%, *n* = 58). Over half of mothers delivering in Kathmandu Valley and Phnom Penh received support from a health worker to correctly position and latch the newborn for breastfeeding.

### 3.6 Multivariable regression analysis

Results of the multivariable regression models are shown in Table 3. Parity was associated with a lower risk of use of BMS after delivery of a newborn in Kathmandu Valley; with each additional child, mothers were 29.0% less likely use BMS. Increasing maternal age had a similar association in both sites, though this relationship was not statistically significant in Phnom Penh. Phnom Penh mothers in the middle (odds ratio: 2.67, 95% CI [1.32, 5.40]) and highest (odds ratio: 3.49, 95% CI [1.08, 11.25]) wealth tercile were both more likely to provide BMS compared with mothers in the lowest wealth tercile. None of the measures of exposure to infant feeding messages or commercial promotions for BMS outside the health system showed a significant association with BMS feeding in delivery facilities in Phnom Penh. In Kathmandu Valley, however, mothers who received friends' or family members' recommendations to use BMS had higher odds of providing their newborn a BMS feed, as compared with mothers who did not receive such a recommendation.

**Table 3:** Unadjusted and adjusted models for use of BMS for newborns among mothers prior to discharge from delivery facilities[Link] ^,^ [Link]

	KATHMANDU VALLEY (N=304)				PHNOM PENH (N=306)			
	Unadjusted		Adjusted		Unadjusted		Adjusted	
	OR	95% CI	OR	95% CI	OR	95% CI	OR	95% CI
**Maternal & household characteristics**								
Age (years)	1.05	1.00 – 1.10	1.12	1.09 – 1.15^***^	1.02	0.98 – 1.08	1.03	0.98 – 1.09
Parity (# of children)	0.44	0.30 – 0.64^***^	0.29	0.21 – 0.39^***^	0.79	0.63 – 1.00	0.79	0.60 – 1.02
*Level of education*								
No education/primary only	0.21	0.10 – 0.45^***^	0.75	0.54 – 1.03	0.40	0.21 – 0.77^**^	0.86	0.30 – 2.44
Secondary	0.30	0.15 – 0.62^**^	1.23	0.63 – 2.38	0.73	0.39 – 1.36	1.02	0.41 – 2.49
Tertiary (reference)	1	‐	1	‐	1	‐	1	‐
*Wealth tercile*								
Lowest tercile (reference)	1	‐	1	‐	1	‐	1	‐
Middle tercile	1.28	0.74 – 2.23	1.04	0.70 – 1.56	2.54	1.41 – 4.56^**^	2.67	1.32 – 5.40^**^
Highest tercile	2.79	1.58 – 4.93^***^	1.02	0.65 – 1.60	4.39	2.29 – 8.41^***^	3.49	1.08 – 11.25^*^
Works outside home	1.99	1.01 – 3.90^*^	1.64	0.60 – 4.50	1.30	0.79 – 2.14	1.16	0.74 – 1.82
Child sex (male)	1.27	0.80 – 2.00	1.39	0.83 – 2.36	0.96	0.61 – 1.51	1.00	0.70 – 1.43
**Exposure to infant feeding messages outside health system**								
Observed BMS promotion in media or retailer	1.16	0.69 – 1.93	2.07	0.74 – 5.77	1.79	0.96 – 3.34	1.59	0.75 – 3.39
Recommendation from peers/family to use BMS	3.22	1.48 – 7.01^**^	9.41	2.83 – 31.22^***^	1.45	0.83 – 2.52	0.97	0.57 – 1.66
Exposure to IYCF educational messages in mass media	2.19	1.18 – 4.06^*^	0.87	0.62 – 1.23	0.99	0.62 – 1.56	0.67	0.37 – 1.22
**Exposure to infant feeding messages inside health system**								
Observed BMS promotion inside a health facility	1.11	0.48 – 2.59	0.84	0.31 – 2.28	1.03	0.65 – 1.62	0.84	0.47 – 1.52
Recommendation from health professional to use BMS	20.33	10.99 – 37.60^***^	24.87	6.05 – 102.29^***^	2.29	1.10 – 4.76^*^	2.42	1.20 – 4.91^*^
Exposure to IYCF educational messages inside health system	0.97	0.54 – 1.74	0.56	0.19 – 1.62	0.64	0.41 – 1.01	0.70	0.37 – 1.34
Received information on breastfeeding during ANC	0.27	0.13 – 0.59^**^	0.26	0.16 – 0.42^***^	0.84	0.52 – 1.34	0.56	0.22 – 1.43
**Characteristics of the delivery**								
Caesarean delivery	12.35	5.89 – 25.89^***^	8.15	1.55 – 42.77^*^	3.13	1.71 – 5.70^***^	2.07	0.56 – 7.69
Early initiation of breastfeeding	0.32	0.20 – 0.51^***^	0.57	0.37 – 0.87^**^	0.28	0.17 – 0.46^***^	0.43	0.19 – 1.01
Held newborn immediately after delivery	0.77	0.34 – 1.72	1.22	0.53 – 2.80	0.42	0.26 – 0.67^***^	0.96	0.37 – 2.46
Discharged before 3 days post‐delivery	0.14	0.07 – 0.26^***^	1.27	0.79 – 2.03	0.33	0.20 – 0.53^***^	0.70	0.33 – 1.35
**Breastfeeding support**								
Received positioning/ attachment assistance for breastfeeding	1.73	1.08 – 2.78^*^	0.87	0.52 – 1.46	0.51	0.32 – 0.82^**^	0.54	0.38– 0.77^**^

*Notes.* Odds ratios and 95% confidence intervals (CI) generated using logistic regression with ^*^p ≤ 0.05 ^**^p ≤ 0.01 ^***^p ≤ 0.001. Maternal age and parity are continuous variables, education level and wealth tercile are categorical, and all other variables are binary categorical.

ANC: antenatal care; BMS: breast milk substitute; CI: confidence interval; IYCF: infant and young child feeding; OR: odds ratio.

In both study populations, receiving a recommendation from a health professional to use BMS was highly associated with BMS feeding before discharge from delivery facilities. Kathmandu Valley and Phnom Penh mothers who received such a recommendation had 24.87 (95% CI [6.05, 102.29]) and 2.42 (95% CI [1.20, 4.91]) times the odds, respectively, of providing a BMS during their stay in the delivery facility, as compared with mothers who did not receive a recommendation from a health professional. Newborns delivered by caesarean section, as compared with newborns from vaginal deliveries, had 8.15 (95% CI [1.55, 42.77]) times the odds of receiving a BMS feed before being discharged from delivery facilities in Kathmandu Valley. Early initiation of breastfeeding was associated with lower use of BMS before discharge in Phnom Penh, reducing the odds by 57%.

## 4 DISCUSSION

This study found a high prevalence of BMS use among newborns prior to discharge from health facilities, and feeding with BMS was strongly associated with receiving a recommendation from a health professional and, to a lesser extent, from a family member. Among a representative sample of mothers delivering healthy, term, singleton infants in Phnom Penh and Kathmandu Valley health facilities, BMS feeding was prevalent and associated with factors both inside and outside the health system. Not all of the assumed covariate variables in the conceptual framework had the expected effect on likelihood of introducing BMS in health facilities after birth. Differences in covariates seen between the two regression models suggests that although the prevalence of BMS feeding was similar in Phnom Penh and Kathmandu Valley, the factors influencing BMS use in the two contexts may vary.

This study adds to the body of literature on factors that are associated with the introduction of BMS in delivery facilities while revealing trends specific to two urban LMIC contexts in South and Southeast Asia. Recommendations from nurses, midwives, doctors, and other health professionals did prove to be highly predictive of BMS use in both study populations. This finding is consistent with other studies that have evaluated determinants of non‐medically indicated prelacteal feeding of BMS (Boban & Zakarija‐Grković, 2016; Grassley et al., 2014; Temple Newhook et al., 2017). Lack of adequate or continued training and support for frontline health care workers could contribute to high rates of BMS feeding in delivery facilities; hospitals with robust breastfeeding education and policies tend to have better breastfeeding outcomes in the first days after birth (Li et al., 2014). There is also a long history of commercial promotion of BMS through solicitation or implicit endorsement of health workers or health facilities (Walker, 2015). Formula companies may seek to influence health professionals to add credibility to their products and drive use through their recommendations (Wright & Waterston, 2006). Health worker attitudes or exposure to such practices was not assessed in this study; as such, this remains a speculative explanation for the behaviour of medical professionals in the two countries.

Findings related to parity and recommendations from friends and family were also consistent with existing literature. First time mothers in Kathmandu Valley had higher odds of providing a BMS in delivery facilities in this study. Higher parity has been associated with timely breastfeeding initiation across Asia (Sharma & Byrne, 2016), whereas first time mothers have been observed to be likelier to introduce prelacteal feeds (Patil et al., 2015) . Encouragement to use BMS from mothers' family and peer groups was an important covariate of BMS feeding among Kathmandu Valley mothers. The strong influence of grandmothers and mothers‐in‐law in particular has been previously noted as impacting mothers' decisions to use BMS as a prelacteal feed in Nepal (Khanal et al., 2013). However, these same family and peer networks could also be leveraged to support behaviour change interventions to improve breastfeeding. Meetings with trained peer facilitators have been shown to modestly improve infant and young child feeding knowledge and practices among mothers in rural areas of Nepal (Singh, Klemm, Mundy, Rana, & Pun, 2017). The relationship between peer recommendations and BMS use was not found in Phnom Penh, further suggesting that the factors that influence newborn feeding are variable and context specific.

Self‐reported observation of commercial promotions for BMS among mothers was not a covariate of BMS use before discharge from delivery facilities. There were substantial differences in the proportion of mothers who reported exposure to BMS promotions in the two sites; almost all Phnom Penh mothers reported observing media and retail promotions (84.6%), and nearly half (45.1%) reported seeing them within health facilities. This is in contrast with the 27.0% and 7.9% of mothers, respectively, in Kathmandu Valley. It is unclear why mothers in the two sites differed so widely in their reported exposure; both Cambodia and Nepal have strong laws restricting such promotion of BMS (WHO, UNICEF, & IBFAN, 2016). If commercial promotions for BMS are as pervasive in Phnom Penh as these mothers' reports suggest, exposure may have been too high to observe differences among mothers who provided BMS to their newborn and those that did not. It is worth noting that the impact of marketing on decision‐making about breastfeeding and BMS use may act in more complex ways than through exposure alone (Parry, Taylor, Hall‐Dardess, Walker, & Labbok, 2013; Sobel et al., 2011), making any relationships difficult to determine in either sample.

Breastfeeding support from health professionals has been shown to be effective in encouraging initiation of breastfeeding and breastfeeding through 6 months of age (Balogun et al., 2016; Jarlenski et al., 2014). Among mothers in this study, concerns about insufficient or delayed milk supply were commonly cited reasons for introducing BMS. The high prevalence of these concerns is also evidenced by a 2017 systematic review conducted across LMIC (Kavle, LaCroix, Dau, & Engmann, 2017). Support and appropriate education on infant behaviour and cues from health professionals can aid in improving breastfeeding outcomes in developing country contexts (Imdad, Yakoob, & Bhutta, 2011; Khanal, Lee, Karkee, & Binns, 2015; Lou et al., 2014). Breastfeeding information and education received before birth during antenatal care visits were inversely associated with introduction of BMS among the relatively small proportion of Kathmandu Valley mothers who received it. No significant effect was observed in Phnom Penh, though many more mothers reported having received such information. Quality and specificity of antenatal breastfeeding education were not evaluated in this study but could have been a limiting factor in Phnom Penh; content gaps in breastfeeding messages and support elsewhere in Cambodia have been suggested as a barrier to successful breastfeeding (Bazzano, Oberhelman, Storck Potts, Taub, & Chivorn, 2015).

An inverse pattern was observed when it comes to health professional support for positioning and attachment for breastfeeding after birth. Roughly equal numbers of mothers in Phnom Penh and Kathmandu Valley reported receiving this support, but it was associated with lower rates of BMS use only in Phnom Penh. Health professionals who provide support for establishing breastfeeding in Kathmandu Valley may not be imparting sufficient information on the importance of early breastfeeding initiation and exclusive breastfeeding. Few mothers in Kathmandu Valley reported holding their infant immediately after giving birth, which in this study served as a proxy for skin‐to‐skin contact between newborn and mother. Immediate skin‐to‐skin contact is a powerful covariate of early breastfeeding initiation and successful exclusive breastfeeding (Lau et al., 2018). Delaying this first skin‐to‐skin contact may contribute to breastfeeding difficulty and the provision of supplemental or replacement feeds of BMS soon after birth in Kathmandu Valley delivery facilities. Only 41% reported initiating breastfeeding within 1 hr after birth, and those who did so were less likely to introduce BMS to their newborn. In Phnom Penh, early initiation was not significantly related to reduced use of BMS in the regression when other variables were taken into account.

The caesarean delivery rates in Phnom Penh and Kathmandu Valley were 24% and 30%, respectively—both high relative to the 10–15% of births deemed acceptable and medically necessary by the World Health Organization (2015). Delivery by caesarean section increased the odds of introducing BMS in Kathmandu Valley. This effect was seen in Phnom Penh but did not reach statistical significance in the regression model when other variables were taken into account. Caesarean delivery is a commonly reported barrier to breastfeeding in LMIC (Kavle et al., 2017; Patel et al., 2015; Takahashi et al., 2017) but is not always found to increase likelihood of early BMS use, especially if the caesarean delivery is an emergency procedure rather than elective (Zanardo et al., 2012). Our survey did not distinguish between emergency and elective or planned caesarean deliveries, which may help explain the absence of association with BMS feeding in Phnom Penh. Early initiation of breastfeeding (regardless of mode of delivery) was associated with lower rates of BMS use in the delivery facility in Kathmandu Valley. Caesarean delivery and subsequent separation of mother and newborn can delay initiation of breastfeeding, but mothers who are able to initiate breastfeeding successfully are equally likely to be exclusively breastfeeding at 6 months as their counterparts who deliver vaginally (Prior et al., 2012). Routine separation of mother–infant dyads after birth removes the opportunity for early initiation of breastfeeding. When facilitated by health professionals, skin‐to‐skin contact after caesarean in particular helps promote breastfeeding initiation (Beake, Bick, Narracott, & Chang, 2017; Chiou, Chen, Yeh, Wu, & Chien, 2014; Moore, Bergman, Anderson, & Medley, 2016; Stevens, Schmied, Burns, & Dahlen, 2014).

This study has several important limitations. First, the cross‐sectional design of this study limits the ability to draw causality between the covariates and outcome of early BMS use. Second, maternal beliefs and perceptions were not included as measurements in this study and therefore not included in the statistical models. Maternal intent or motivation to breastfeed can be powerful factors in initiating and sustaining breastfeeding (Aghdas, Talat, & Sepideh, 2014; Loke & Chan, 2013; Wu, Hu, McCoy, & Efird, 2014). Additionally, maternal perceptions of BMS use, often influenced by advertising and social norms, were not assessed in this study. These are important intermediate factors in the conceptual framework, which could have influenced the results and the number of hypothetical covariates, which were shown to be associated with BMS feeding. Third, this study relied on maternal self‐reporting; recall bias may have occurred for questions assessing maternal exposure to commercial promotion of BMS and infant feeding messaging during pregnancy. Phrasing of survey questions may also have limited the ability to draw conclusions from mothers' self‐reported data—particularly in regard to “holding the infant immediately after birth,” an imperfect proxy for skin‐to‐skin contact. Finally, as interviews were conducted upon hospital discharge, mothers reported on their infant feeding practices over varying lengths of time. Mothers generally had longer stays after delivery in Phnom Penh, where 63.7% were discharged after 3+ days. In contrast, 31.6% of Kathmandu Valley mothers were discharged after 3+ days, and 56.9% were discharged between 1 and 2 days after birth. This could have provided Phnom Penh mothers more opportunity to introduce BMS or, conversely, greater exposure time for breastfeeding counselling or advice from health professionals. Length of stay after delivery varies across the world, and evidence suggests it is less than the recommended 24 hr in many countries (Campbell, Cegolon, Macleod, & Benova, 2016). National or regional data on length of stay in health facilities are not available for urban Nepal and Cambodia (Campbell, Cegolon, et al., 2016), so it is unclear whether the self‐reported time frames were the norm, and if these results are generalizable beyond Kathmandu Valley and Phnom Penh.

## 5 CONCLUSIONS AND RECOMMENDATIONS

Immediate skin‐to‐skin contact and initiation of breastfeeding within 1 hr of birth are key interventions for supporting optimal breastfeeding practices through infancy (Debes et al., 2013; World Health Organization, 2013). Hospital policy and practices can play a key role in supporting and facilitating these behaviours and in avoiding unnecessary early BMS supplementation. Better breastfeeding support in health facilities may be necessary to improve breastfeeding rates in urban Cambodia and Nepal. However, breastfeeding promotion and education must target not only mothers giving birth but also family and peer groups who may influence infant feeding decisions. Given the potential influence of friends and family members on decision‐making about newborn feeding, peer mobilization strategies may be an avenue through which breastfeeding messages can be communicated to expecting mothers, especially in Kathmandu Valley.

Health professional recommendations and support have a powerful influence on BMS feeding before discharge from delivery facilities in Kathmandu Valley and Phnom Penh. This represents an opportunity to train health professionals to correctly identify and support mothers with difficulty of breastfeeding and reduce the need for introduction of supplementary BMS feeds. More research is needed on why BMS is recommended to healthy term infants.

Special care is needed for mothers delivering by caesarean to enable them to initiate and sustain breastfeeding. Further research is needed on how to promote immediate initiation of breastfeeding after caesarean delivery in a safe and culturally appropriate manner in these contexts.

BMS promotion is taking place in health facilities throughout urban Phnom Penh and Kathmandu Valley, despite the strong laws in each county. Legislation designed to curb the influence of commercial marketing is key to ensuring informed decision‐making around the foods an infant receives in their first days of life. Both Cambodia and Nepal have the potential to improve practices and safeguard the health of the next generation.

## CONFLICTS OF INTEREST

The authors declare that they have no conflicts of interest.

## CONTRIBUTIONS

MC oversaw questionnaire development, adapted the conceptual framework, and prepared the manuscript. SH and EZ conceptualized the study design and oversaw implementation. AP oversaw data collection, data management, and conducted the statistical analysis. All authors reviewed and provided inputs on manuscript drafts.
